# Epidemiology and clinical characteristics of patients discharged from the ICU in a vegetative or minimally conscious state

**DOI:** 10.1371/journal.pone.0253225

**Published:** 2021-06-25

**Authors:** Piotr Knapik, Dawid Borowik, Daniel Cieśla, Ewa Trejnowska

**Affiliations:** 1 Department of Anesthesiology, Intensive Therapy and Emergency Medicine, Silesian Centre for Heart Diseases, Medical University of Silesia, Zabrze, Poland; 2 Department of Science and New Technologies, Silesian Centre for Heart Diseases, Zabrze, Poland; Heidelberg University Hospital, GERMANY

## Abstract

**Purpose:**

A significant percentage of patients are discharged from intensive care units (ICU) with disorders of counciousness (DoC). The aim of this retrospective, case-control study was to compare patients discharged from the ICU in a vegetative state (VS) or minimally conscious state (MCS) and the rest of ICU survivors, and to identify independent predictors of DoC among ICU survivors.

**Methods:**

Data from 14,368 adult ICU survivors identified in a Silesian Registry of Intensive Care Units (active in the Silesian Region of Poland between October 2010 and December 2019) were analyzed. Patients discharged from the ICU in a VS or MCS were compared to the remaining ICU survivors. Pre-admission and admission variables that independently influence ICU discharge with DoC were identified.

**Results:**

Among the 14,368 analyzed adult ICU survivors, 1,064 (7.4%) were discharged from the ICU in a VS or MCS. The percentage of patients discharged from the ICU with DoC was similar in all age groups. Compared to non- DoC ICU patients, they had a higher mean APACHE II and SAPS III score at admission. Independent variables affecting ICU discharge with DoC included unconsciousness at ICU admission, cardiac arrest and craniocerebral trauma as primary cause of ICU admission, as well as a history of previous chronic neurological disorders and cerebral stroke (*p*<0.001).

**Conclusion:**

Discharge in a VS and MCS was relatively frequent among ICU survivors. Discharge with DoC was more likely among patients who were unconscious at admission and admitted to the ICU due to cardiac arrest or craniocerebral trauma.

## Introduction

The widespread popularization of cardiopulmonary resuscitation, rapid development of intensive care and the increasing invasiveness of medical procedures tend to generate new medical, social and ethical problems [1]. In the intensive care unit (ICU), we tend to observe a substantial number of patients who, after emerging from life-threatening conditions, present symptoms of disorders of counciousness (DoC) [2]. DoC are known to be more frequent in the aging population and the aging population is known to increase the need for intensive care unit (ICU) beds [3]. This will have a significant impact on healthcare systems, as ICU patient numbers are growing worldwide [4–6].

Patients with DoC may initially remain in a coma due to anoxemic encephalopathy, but this could be also associated to traumatic and vascular etiology. Over time, their condition begins to evolve in different directions. The ultimate effect of treatment can be extremely unfavorable (cerebral death, VS or MCS) or satisfactory (full recovery–Cerebral Performance Category 1 or 2), with a broad spectrum of varying degrees of dysfunction to the central nervous system that fall between these two extremes [2,7–9]. Patients with neurological damage that is profound and takes the form of VS or MCS, are particularly challenging for ICU teams [10–12].

Interestingly, epidemiological data for patients with DoC at ICU discharge seem to be missing in the medical literature worldwide. Moreover, this population has never been studied in Poland, where ICU mortality is known to be excessively high [13]. The reason for the excessive mortality seems to be relatively simple—in many Polish hospitals, patient’s death is still difficult to be accepted outside the ICU area [14]. In such circumstances, patients discharged from the ICU with DoC are of particular importance, as they contribute to the subset of patients with unfavorable outcomes. This creates an additional psychological and emotional burden for ICU staff.

The primary aim of our study was to compare patients discharged from the ICU with DoC with the other ICU survivors. The secondary aim was to identify independent predictors of ICU discharge with DoC.

## Patients and methods

We conducted a retrospective, case-control, multicenter study involving patients hospitalized in general ICUs in the Silesian Region of Poland between October 1, 2010 and December 31, 2019. Specialized ICUs operating in the Silesian Region have been excluded from reporting to the Registry. Overall, there were 25,416 hospitalizations identified in Silesian Registry of Intensive Care Units at that time, with 11,048 hospitalizations resulting in ICU death (43.5%). Data from the remaining 14,368 hospitalizations of ICU survivors were analyzed. This voluntary registry collected information on the health burden of patients admitted to ICUs, their general condition on admission, causes of the disease as well as the course and results of ICU treatment [15]. Data were entered into the registry by ICU physicians or medical secretaries working directly under the supervision of ICU physicians. Approximately 35% of all Silesian ICUs reported their data to the registry. The registry does not contain any personal data. Due to the retrospective and anonymous nature of the study, the Ethical Committee at the Medical University of Silesia in Katowice waived the need for obtaining the consent of patients to participate in the study.

Patients discharged from the ICU in a VS or MCS [11,12] were identified in the registry and their data were compared to ICU survivors without these types of DoC. The factors that were compared included demographic parameters, comorbidities, severity of general condition at time of ICU admission, main reasons for ICU admission and the course of ICU treatment. For the aim of the multivariate regression analysis, variables with missing data (<5%) were defaulted to the most common value present in the majority of cases.

Analyses were performed using Dell Statistica, version 13 (Dell Inc. 2016). Demographic data were presented using descriptive statistical methods and compared using the Student’s t-test or Mann-Whitney test. The choice of test was dependent on the result of the Kolmogorov-Smirnov test. For comparison of qualitative variables, the Chi-square test with Yates correction was used.

Independent pre-admission and admission variables that might influence discharge with DoC (defined as VS or MCS) were identified on the basis of the data presented in Tables 1 and 2 and Fig 2, supplemented with information on whether a patient was unconscious, intubated and mechanically ventilated at ICU admission. The effect of independent variables on the outcome variable of interest was calculated by means of univariate logistic regression. Variables with a *P* value <0.05 were included in multivariate logistic regression analysis.

**Table 1:** Medical status at ICU admission among ICU patients discharged from the ICU with or without DoC.

Group of	Variables	DoC		No DoC		*P*
variables		(n = 1,064)		(n = 13,304)		
Admission	First	997	(93.7%)	12,460	(93.7%)	0.996
	Second	60	(5.6%)	730	(5.5%)	0.889
	Another	7	(0.7%)	114	(0.9%)	0.611
Co-morbidities	Coronary artery disease	395	(37.1%)	4,872	(36.6%)	0.768
	Heart failure	277	(26.0%)	3,959	(29.8%)	**0.011**
	Arterial hypertension	486	(45.7%)	6,853	(51.5%)	**<0.001**
	Disseminated atherosclerosis	291	(27.4%)	3,611	(27.1%v	0.912
	Chronic respiratory failure	87	(8.2%)	1,735	(13.0%)	**<0.001**
	Home oxygen therapy	6	(0.6%)	211	(1.6%)	**0.012**
	Extreme obesity	46	(4.3%)	764	(5.7%)	0.063
	Cachexia	25	(2.4%)	384	(2.9%)	0.359
	Alcoholism	143	(13.4%)	1,050	(7.9%)	**<0.001**
	Diabetes	225	(21.2%)	3,137	(23.6%)	0.077
	Chronic renal failure	86	(8.1%)	1,563	(11.8%)	**<0.001**
	Dialysis dependency	6	(0.6%)	135	(1.0%)	0.203
	Previous cerebral stroke	101	(9.5%)	864	(6.5%)	**<0.001**
	Chronic neurological disorders	132	(12.4%)	1,030	(7.7%)	**<0.001**
	Systemic autoimmune diseases	8	(0.8%)	154	(1.2%)	0.291
	Post transplant	1	(0.1%)	30	(0.2%)	0.585
	Cancer	10	(0.9%)	1,425	(10.7%)	**<0.001**
	Pregnancy	0	(0.0%)	37	(0.3%)	0.159
	None	130	(12.2%)	1,555	(11.7%)	0.640

**Table 2:** Primary reason for ICU admission among patients discharged from the ICU with or without DoC.

Variables	DoC		No DoC		*P*
	(n = 1,064)		(n = 13,304)		
Acute respiratory failure	724	(68.1%)	9,458	(71.1%)	**0.039**
Exacerbation of respiratory failure	55	(5.2%)	1,206	(9.1%)	**<0.001**
Shock	203	(19.1%)	2,729	(20.5%)	0.281
Exacerbation of circulatory failure	388	(36.5%)	5,159	(38.8%)	0.145
Multiorgan failure	60	(5.6%)	950	(7.1%)	0.075
Cardiac arrest	526	(49.4%)	1,729	(13.0%)	**<0.001**
Disorders of consciousness	490	(46.1%)	4,314	(32.4%)	**<0.001**
Postoperative	140	(13.2%)	5,034	(37.8%)	**<0.001**
Multiple trauma	45	(4.2%)	627	(4.7%)	0.520
Craniocerebral trauma	132	(12.4%)	576	(4.3%)	**<0.001**
Acute pancreatitis	2	(0.2%)	174	(1.3%)	**0.002**
Obstetric complications	0	(0.0%)	81	(0.6%)	**0.019**
Acute neurological disorders	153	(14.4%)	881	(6.6%)	**<0.001**
Intoxication	20	(1.9%)	263	(2.0%)	0.917
Severe metabolic disorders	44	(4.1%)	449	(3.4%)	0.221
Bacterial infection	120	(11.3%)	2,332	(17.5%)	**<0.001**
Viral infection	8	(0.8%)	57	(0.4%)	0.202
Sepsis	20	(1.9%)	744	(5.6%)	**<0.001**

The multivariate model was fitted using the stepwise method, where *p*<0.05 was set as inclusion and removal criteria. For purposes of all calculations, statistical significance was accepted at a level of *P*<0.05.

## Results

Overall, there were 25,416 hospitalizations identified in Silesian Registry of Intensive Care Units at that time, with 11,048 hospitalizations resulting in ICU death (43.5%). Data from the remaining 14,368 hospitalizations of ICU survivors were analyzed.

**Fig 1:**
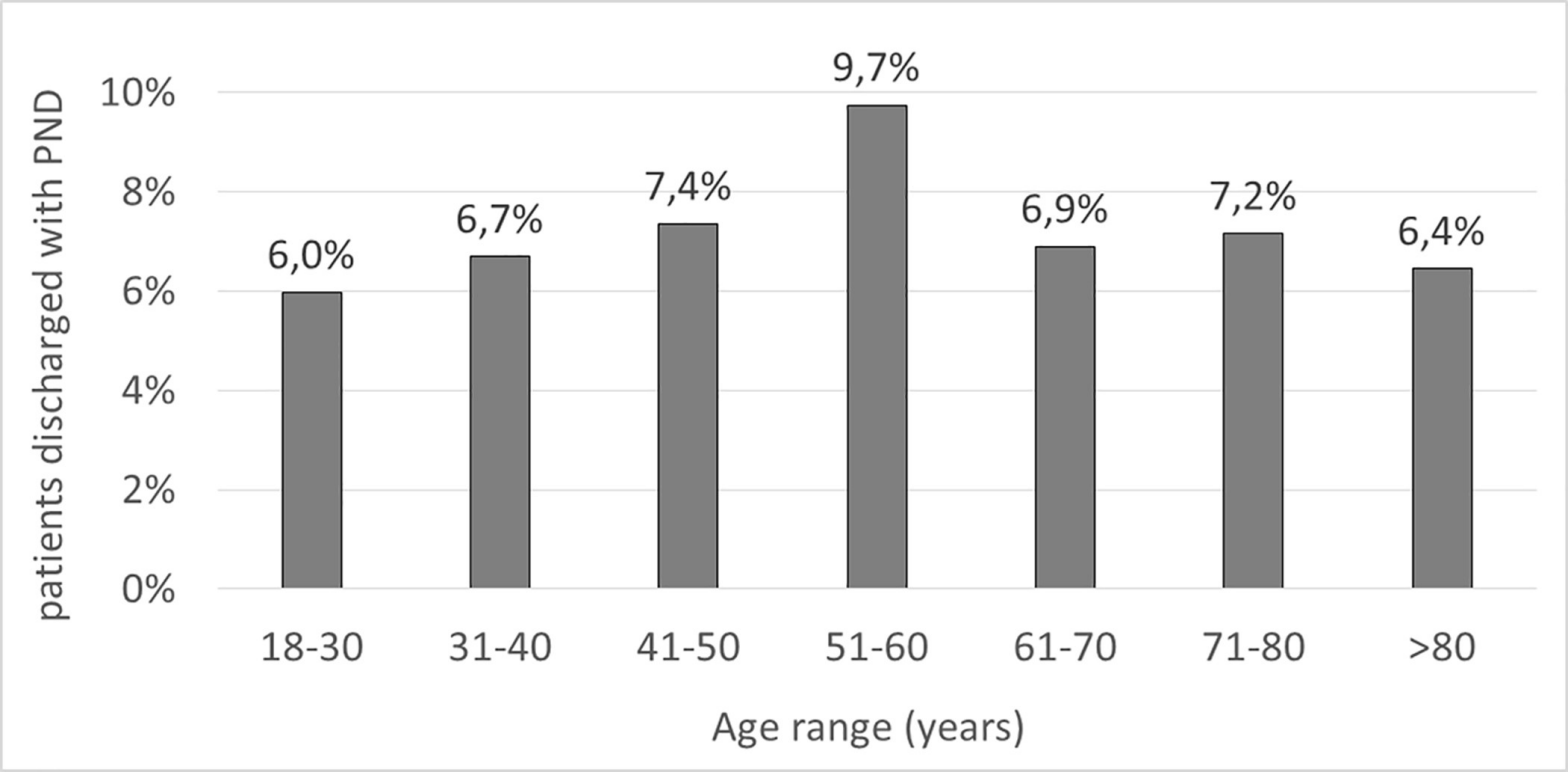
Percentage of patients discharged with DoC among ICU survivors stratified by age.

Among 14,368 analyzed survivors of ICU stay, 1,064 patients (7.4%) were discharged from the ICU with a DoC. The percentage of patients discharged with DoC was broadly similar across consecutive age ranges, with an exception for the age range of 51–60 years where the percentage of patients with DoC among ICU survivors was as high as 9.7% **(Fig 1).**

Comparison of basic demographic data between patients discharged with a DoC and non- DoC ICU survivors revealed that there were more males among patients discharged in a VS or MCS (63.1% vs 57.7%, *p* = 0.001). The mean age in both groups was similar: 61.6 ±15.3 years among patients discharged with DoC and 62.0 ±16.3 years in the non- DoC group (*p* = 0.459).

**Fig 2:**
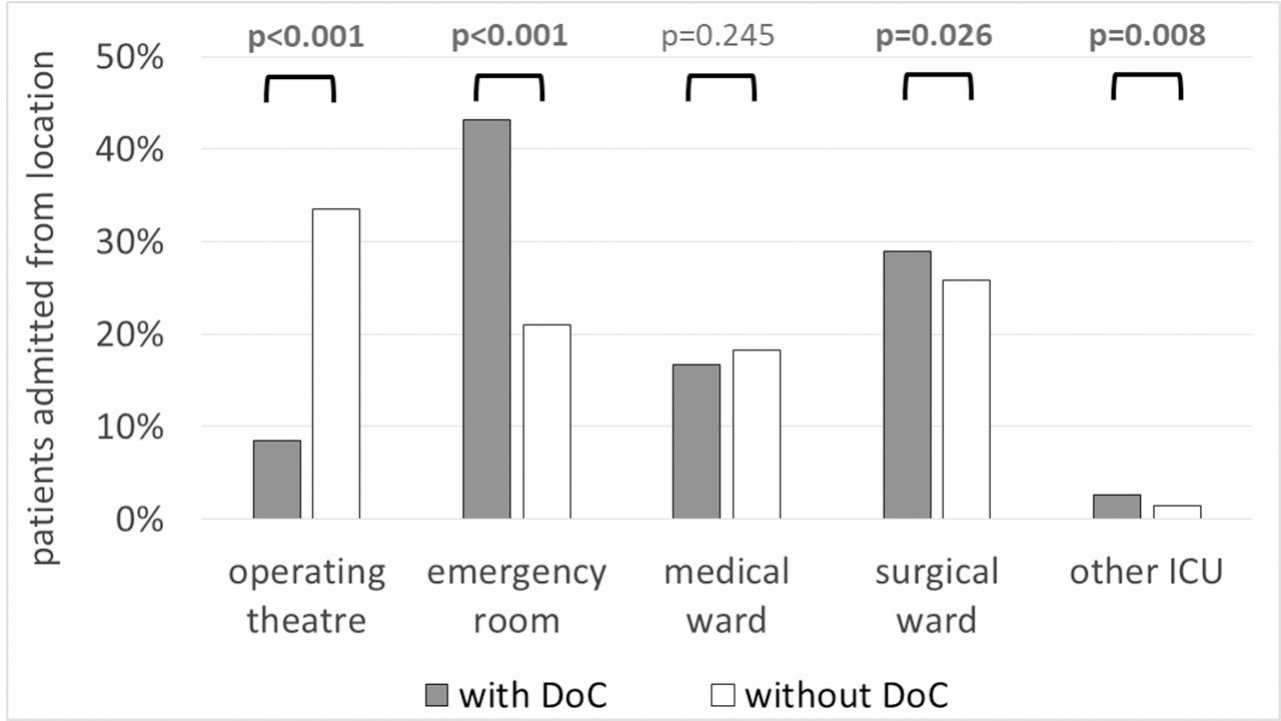
Sources of ICU admission among patients discharged from the ICU with and without DoC.

Patients discharged with DoC were mainly admitted to the ICU from the emergency department or directly from the place of event (43.2%). The distribution of sources of admission among these patients was generally different from the non- DoC ICU population **(Fig 2)**.

In regard to comorbidities, patients discharged from the ICU with DoC were found to more frequently have past cerebral stroke, chronic neurological disorders and alcohol dependency. On the other hand, their health status was less frequently burdened with chronic diseases such as chronic respiratory failure, home oxygen therapy, chronic renal failure and cancer **(Table 1)**.

ICU admission scoring systems were only available for 51.2% of patients for the APACHE II (Acute Physiology and Chronic Health Evaluation II) [16] score and 26.5% of patients for the SAPS III (Simplified Acute Physiology Score III) score [17], with comparable percentages among patients discharged from the ICU with and without DoC. Patients discharged from the ICU with DoC had a higher mean APACHE II and SAPS III score at admission in comparison to the other ICU survivors (23.4 ±7.1 vs 19.4 ±7.9 points, *p*<0.001 and 52.1 ±15.1 vs 36.1 ±16.9 points, *p*<0.001, respectively).

ICU survivors discharged with DoC were more frequently admitted to the ICU due to cardiac arrest (49.4% vs 13.0%), craniocerebral trauma (12.4% vs 4.3%), disorders of consciousness (46.1% vs 32.4%,) or acute neurological disorders (14.4% vs 6.6%) **(Table 2)**. In contrast, patients discharged with DoC were less frequently admitted to the ICU following elective or emergency surgical procedures (13.2% vs 37.8%), bacterial infection (11.3% vs 17.5%) and sepsis (1.9% vs 5.6%). These patients were also more frequently unconscious (91.7% vs 57.5%), intubated (87.7% vs 71.3%) and mechanically ventilated (86.8% vs 68.5%) at ICU admission with a lower mean Glasgow Coma Scale Score on ICU admission (4.8 ±2.7 vs 8.8 ±4.6 points, respectively). All of these differences were statistically significant (*p*<0.001).

During ICU stay, patients discharged with DoC were more likely to have been invasively ventilated and undergone tracheostomy (88.6% vs. 77.4%, *p*<0.001 and 64.8% vs. 12.3%, *p*<0.001, respectively). Patients in this group were also more frequently treated with therapeutic hypothermia (3.8% vs 0.6%, *p*<0.001). The full details describing treatment procedures in both groups of patients are shown in **Table 3**.

**Table 3:** ICU treatment in patients discharged from the ICU with or without DoC.

Variables	DoC		No DoC		*P*
	(n = 1,064)		(n = 13,304)		
Catecholamines	740	(69.6%)	8,001	(60.1%)	**<0.001**
Intubation	669	(62.9%)	7,957	(59.8%)	0.053
Tracheostomy	689	(64.8%)	1,632	(12.3%)	**<0.001**
Invasive ventilation	943	(88.6%)	10,293	(77.4%)	**<0.001**
Non-invasive ventilation	15	(1.4%)	911	(6.9%)	**<0.001**
Renal replacement therapy	52	(4.9%)	824	(6.2%)	0.100
Operation while in the ICU	110	(10.3%)	1,024	(7.7%)	**0.003**
Therapeutic hypothermia	40	(3.8%)	82	(0.6%)	**<0.001**
Intra-aortic balloon pump	16	(1.5%)	215	(1.6%)	0.878
ECMO	6	(0.6%)	32	(0.2%)	0.096

**Fig 3:**
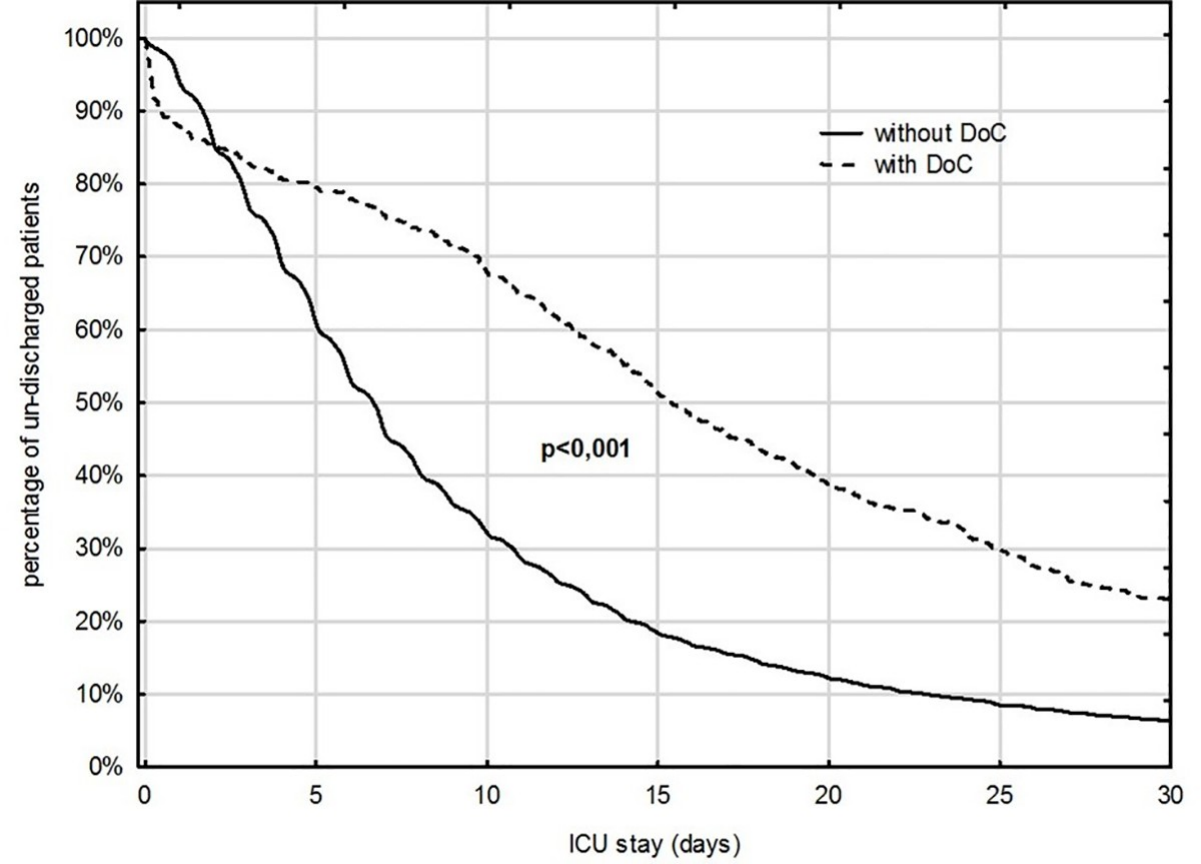
Kaplan-Meier curves presenting discharge curves in patients discharged from the ICU with and without DoC.

The mean time of ICU stay was approximately two times longer among patients discharged with DoC in comparison to non- DoC ICU survivors (21.3 ±21.7 vs 10.5 ±13.7 days, *p*< 0.001). This difference is depicted in a Kaplan-Meier curve (**Fig 3)**.

**Fig 4:**
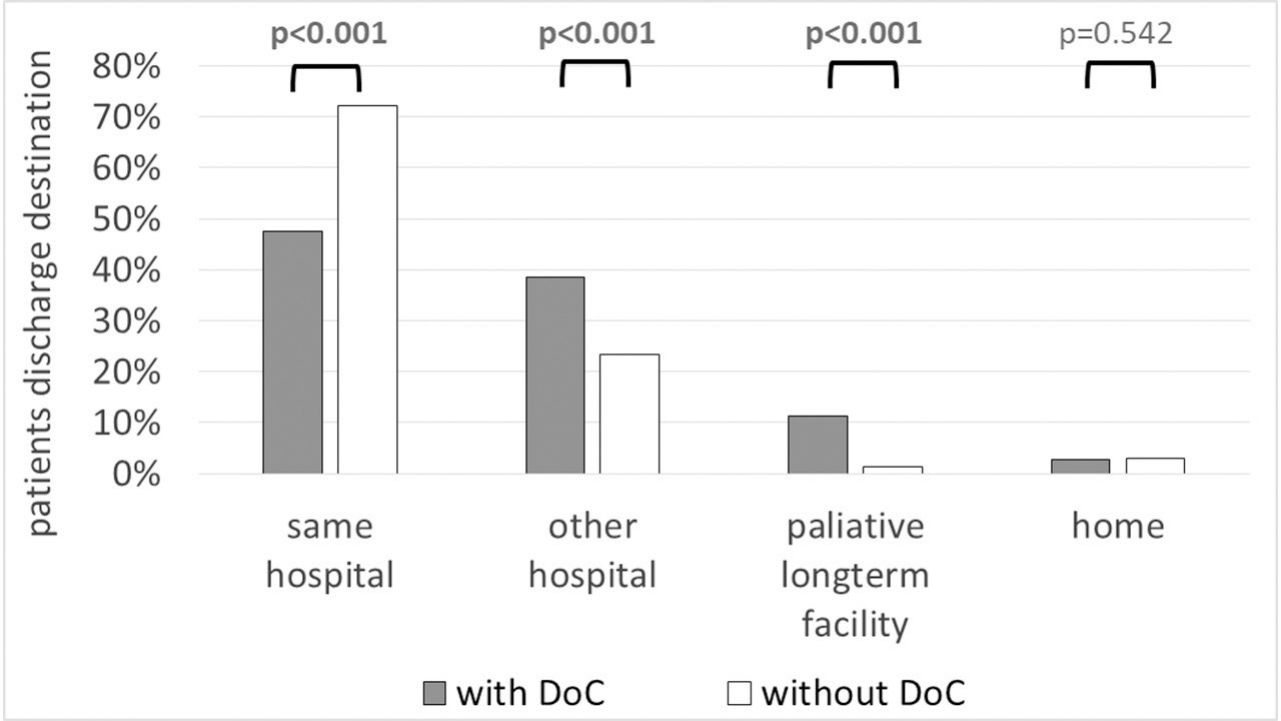
Discharge destinations among ICU patients who completed their ICU stay with and without DoC.

Patients with DoC were more frequently discharged to other hospitals (38.4% vs 23.4%, *p*<0.001) or directly to long-term care facilities (11.3% vs 1.4%, *p*<0.001). Discharge destinations of patients in both groups are presented in **Fig 4.**

**Fig 5:**
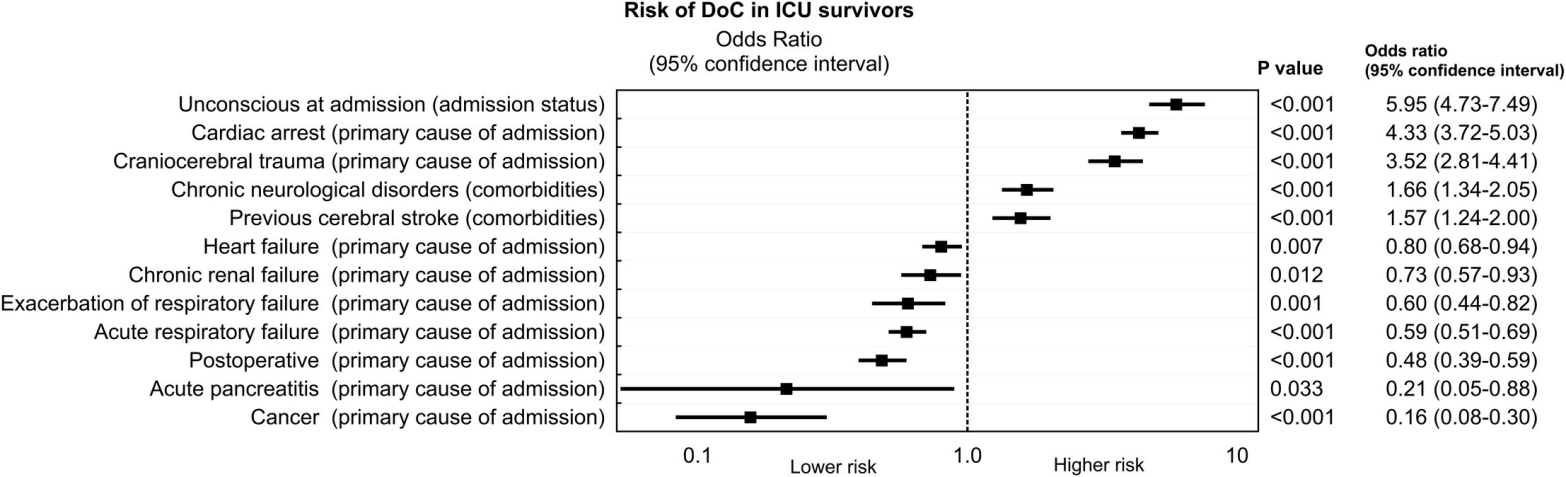
Multivariate analysis–independent predictors of discharge in a DoC.

Independent variables affecting ICU discharge with DoC (*p*<0.001) included unconsciousness at ICU admission (OR 5.9; 95% CI: 4.7–7.5), cardiac arrest and craniocerebral trauma as primary cause of ICU admission (OR 4.3; 95% CI: 3.7–5.0 and OR 3.5; 95% CI: 2.8–4.4, respectively), as well as chronic neurological disorders and previous cerebral stroke among comorbidities (OR 1.7; 95% CI: 1.3–2.1 and OR 1.6; 95% CI: 1.2–2.0, respectively). Several independent variables (known at ICU admission) also affected the lack of DoC at ICU discharge. The full list of independent variables influencing neurological status at ICU discharge is presented in **Fig 5**. All details of the multivariate logistic regression model are presented as the supplement file (S1 File).

## Discussion

In our study, we confirmed that discharge with DoC was relatively frequent among ICU survivors in the Silesian region of Poland. Discharge with DoC most likely occurred among patients who were unconscious at admission and admitted to the ICU due to cardiac arrest or craniocerebral trauma. Unfavorable neurological outcome was also more likely in patients with a history of stroke or chronic neurological disorders.

We analyzed data from 14,386 adult ICU survivors of which 1,064 were discharged from the ICU in a VS or MCS. To our knowledge, there is no study to date in the medical literature that has used similar methodology and comparable sample size. Researchers in this area are instead concentrating on mid-term or long-term follow-up outcomes in these patients, or assessing the effectiveness of professional rehabilitation programs [18–24].

The only study in which researchers aimed to identify independent risk factors at hospital discharge was in patients with traumatic brain injury. The authors of this study however, were analyzing risk factors for a combined unfavorable outcome of both death and VS [25].

The percentage of patients with DoC was the highest in the age range of 51 to 60 years. This may be explained by the fact, that the highest percentage of ICU admissions due to cardiac arrest are observed in this age group, and nearly half of ICU survivors presenting with DoC at ICU discharge previously had cardiac arrest before ICU admission.

The percentage of patients discharged with DoC among ICU survivors in our study was 7.4%. It is difficult to compare this result to other studies. This figure appears to be high; however, it is even higher among patients admitted to long-term geriatric facilities where, according to a report, a clinical diagnosis of VS was made for 15% of patients upon admission [26]. On the other hand, the percentage of patients discharged with VS among patients with traumatic brain injury was only 3.6% [25].

Nearly half of all patients in our study, discharged from the ICU with DoC had been admitted to the ICU due to cardiac arrest, while among ICU survivors with good neurological outcome, the corresponding value was as low as 13%. Despite extensive media coverage of “miracle recoveries” [27], poor-quality survival in these patients is common. It is known that following in-hospital cardiac arrest, only about 18% of patients survive to discharge. These outcomes are even worse among patients who experience out-of-hospital cardiac arrest, where only 2% to 9% of patients survive to discharge [28].

The long-term outcomes of patients with VS and MCS are important. Many of these patients improve slightly over time [29]; however, in the short-term, this is not as apparent. In one report, it was shown that within 2 months of admission in a specialized intensive neurorehabilitation unit, 3.9% of patients admitted with VS or MCS were dead, 35.5% had a full recovery of consciousness while 66.7% remained in VS or MCS [24]. It has also been shown that there are no significant differences in the rehabilitation outcomes of patients with non-traumatic brain injury compared with patients with traumatic brain injur [30,31].

DoC at ICU discharge is a possible outcome not only following cardiac arrest or traumatic brain injury. Long-term cognitive impairment is also frequent after acute respiratory distress syndrome [32]. Additionally, it is important to note that ICU admission may be preceded by profound hypoxia, hypoperfusion or metabolic abnormalities. Therefore, the risk of permanent anoxemic neurological injury is sometimes very high in the period preceding admission to the intensive care area [2].

Due to these reasons, early identification of patients with DoC is of great importance and should be done efficiently [33]. A poor neurological status remains a strong predictor of poor outcome, both at hospital discharge and at one year [34]. An interesting and emerging area is the use of biomarkers for identifying neurological damage, with plasma S100 protein and neuron-specific enolase being the most promising biomarkers in the field [2].

Although we were not able to find a lot of comparative data, it should be noted that the results obtained in this multicenter study conducted across Polish ICUs might differ from results in other countries. These differences relate to the recent correspondence with the Editor of Intensive Care Medicine on excessive mortality in Polish ICUs [11,30]. This issue has been already discussed in detail in our previous study from the Silesian ICU Registry, concentrating on ICU admissions of young adults in Poland [35].

The concept of advanced directives is unknown in Poland; however, since 2014, we have official guidelines issued by the Polish Society of Anesthesiology and Intensive Therapy regarding the ineffective maintenance of organ functions (futile therapy) in ICU patients incapable of giving informed statements of will [36]. Unfortunately, there is no data in the Silesian ICU Registry indicating how many patients with DoC have been treated according to the do-not-resuscitate (DNR) orders described in these guidelines. In a study by Patel et al., the authors aimed to explore the effect of the presence and timing of DNR order on short-term clinical outcomes (including mortality). After propensity score matching, the DNR group had significantly longer stays (9.7 vs 6.0 days, *p*<0.001), was more likely to be discharged to hospice (6.5% vs 0.7%, *p*<0.001) and to die (12.2% vs 0.8%, *p*<0.001). Interestingly, the timing of DNR orders was also important, as patients with early DNR (<24 hours from ICU admission) were less likely to spend time in intensive care, receive a palliative care consultation or die in hospital, and more likely to be discharged home [37]. Patients with DNR orders (more likely to have DoC) were also found to be significantly older and had more comorbidities in comparison to the no-DNR group [37]. Additionally, in our study we were also able to show clear trend to discharge patients with DoC to another hospitals, instead of treating them in the other medical or surgical departments of the same hospital where they have been previously treated in the ICU (Fig 4).

In our study, patients with DoC had higher mean APACHE II and SAPS III scores at admission compared with non-DoC ICU survivors. Patients discharged with DoC were much more likely to be admitted to the ICU due to cardiac arrest (49.4% vs 13.0%, *p*<0.001) and therefore, were more frequently treated with therapeutic hypothermia (3.8% vs 0.6%, *p*<0.001). Generally, DoC is relatively frequent among survivors of cardiac arrest. In a study by Matthews et al., the majority of patients were treated with therapeutic hypothermia (with a target temperature range of 32 to 34 ^o^C) and among those who survived to day 7, 25% had a good neurological recovery (CPC score 1–2), 31% survived to hospital discharge with poor neurologic recovery (CPC score 3–4) and 44% died [34].

The mean time of ICU stay in our study was confirmed to be much longer in patients discharged with DoC. The reasons for this are probably complex. Despite the fact that the Polish Society of Anesthesiology and Intensive Therapy published guidelines on futile therapy already 6 years ago [36], these guidelines are still not widely implemented in Poland. This issue has been already described elsewhere [38].

This problem does not only concern Poland. It has been recently shown that adequate palliative care may be introduced with great heterogeneity and delay in other countries as well [39]. As a result, many patients with severe DoC remain in the ICU and are sometimes actively treated. In Poland it is also not easy to arrange a transfer for these patients from the ICU to the few long-term facilities available [38]. Patients must either give consent for such a transfer or consent has to be issued by the appropriate court. Unfortunately, no fast-track process has been created for these patients in Polish law. Our results clearly indicate the net effect of these problems. Needless to say, these situations create an unnecessary financial burden for the Polish healthcare system. This issue is of even more relevance and importance in the current pandemic situation [40]. Our study has several limitations, including an incomplete representativeness of the sample cohort (i.e. not all Silesian ICUs report to the Silesian ICU Registry). We had no follow-up data for these patients and therefore, our analysis was restricted only to the ICU stay. This is a retrospective study, based on Registry data. Therefore, we were strictly limited to the data available in the Registry and we could not be sure that the diagnosis of VS and MCS was based on a standardized procedures. We also did not have data on time of use of sedatives drugs, time of mechanical ventilation and clinical state at ICU admission. Despite this, the strengths of the study include the uniqueness and importance of the presented data, a large population sample and a relatively broad representation of ICUs.

## Conclusions

We confirmed that discharge with severe DoC (in a VS or MCS) is relatively frequent among ICU survivors in Poland. Discharge with DoC is more likely in patients who are unconscious at admission and admitted to the ICU due to cardiac arrest or craniocerebral trauma. All possible measures should be taken to shorten the ICU stay of these patients once DoC is confirmed.

## Supporting information

S1 File Details of the multivariate logistic regression model. (DOC)

## References

[1] Lamas D (2019) Chronic critical illness. N Engl J Med 370:175–177. 10.1056/NEJMms1310675. 24401058

[2] Knapik P, Knapik M, Partyka R, Broll I, Cieśla D, Wawrzyńczyk M, et al (2016) Utility of serum concentration of protein S100 at admission to the medical intensive care unit in prediction of permanent neurological injury. Kardiochir Torakochirurgia Pol 13:347–352. doi: 10.5114/kitp.2016.64879 28096833 PMC5233766

[3] Atramont A, Lindecker-Cournil V, Rudant J, Tajahmady A, Drewniak N, Fouard A, et al (2019) Association of Age With Short-term and Long-term Mortality Among Patients Discharged From Intensive Care Units in France. JAMA Netw Open. 2:e193215. doi: 10.1001/jamanetworkopen.2019.3215 31074809 PMC6512465

[4] Society of Critical Care Medicine (2020) Critical Care Statistics https://www.sccm.org/Communications/Critical-Care-Statistics. Accessed 9 November 2020.

[5] A Berkshire Hathaway Company (2020) Insights on the Worldwide ICU Beds Industry to 2030 –Featuring Invacare, Paramount Bed Holdings & Medline Industries Among Others. https://www.businesswire.com/news/home/20200420005718/en/Insights-Worldwide-ICU-Beds-Industry-2030 Accessed 9 November 2020.

[6] Prin M, Wunsch H (2012) International comparisons of intensive care: informing outcomes and improving standards. Curr Opin Crit Care. 18: 700–706. doi: 10.1097/MCC.0b013e32835914d5 22954664 PMC3551445

[7] Laureys S, Owen AM, Schiff ND (2004) Brain function in coma, vegetative state, and related disorders. Lancet Neurol. 3:537–546. doi: 10.1016/S1474-4422(04)00852-X 15324722

[8] Provencio JJ, Hemphill JC, Claassen J, et al (2020) The Curing Coma Campaign: Framing Initial Scientific Challenges-Proceedings of the First Curing Coma Campaign Scientific Advisory Council Meeting. Neurocrit Care. 33:1–12. doi: 10.1007/s12028-020-01028-9 32578124 PMC7392933

[9] Faymonville ME, Pantke KH, Berré J, Sadzot B, Ferring M, de Tiège X, et al (2004) Cerebral functions in brain-damaged patients. What is meant by coma, vegetative state, minimally conscious state, locked-in syndrome and brain death? Anaesthesist. 53:1195–1202. doi: 10.1007/s00101-004-0747-4 15597160

[10] Rohaut B, Eliseyev A, Claassen J (2019) Uncovering Consciousness in Unresponsive ICU Patients: Technical, Medical and Ethical Considerations. Crit Care. 23:78. doi: 10.1186/s13054-019-2370-4 30850022 PMC6408788

[11] Multi-Society Task Force on PVS (1994) Medical aspects of the persistent vegetative state. N Engl J Med. 330:1499–1508. doi: 10.1056/NEJM199405263302107 7777031

[12] Giacino JT, Ashwal S, Childs N, Cranford R, Jennett B, Katz DI, et al (2002) The minimally conscious state: definition and diagnostic criteria. Neurology. 58:349–353. doi: 10.1212/wnl.58.3.349 11839831

[13] Weigl W, Adamski J, Goryński P, Kański A, Hultström M (2017) Mortality Rate Is Higher in Polish Intensive Care Units Than in Other European Countries. Intensive Care Med. 43:1430–1432. doi: 10.1007/s00134-017-4804-2 28484784

[14] Knapik P, Knapik M, Trejnowska E, Kłaczek B, Śmietanka K, Cieśla D, et al (2019) Should we admit more patients not requiring invasive ventilation to reduce excess mortality in Polish intensive care units? Data from the Silesian ICU Registry. Arch Med Sci. 15:1313–1320. doi: 10.5114/aoms.2019.84401 31572479 PMC6764313

[15] Krzych ŁJ, Czempik PF, Kucewicz-Czech E, Knapik P (2017) Silesian Registry of Intensive Care Units. Anaesthesiol Intensive Ther. 49:73–75. doi: 10.5603/AIT.2017.0011 28362034

[16] Knaus WA, Draper EA, Wagner DP, Zimmerman JE (1985) APACHE II: A severity of disease classification system. Crit Care Med. 13: 818–829. 3928249

[17] Moreno RP, Metnitz PH, Almeida E, Jordan B, Bauer P, SAPS 3 Investigators et al (2005) SAPS 3-From evaluation of the patient to evaluation of the intensive care unit. Part 2: Development of a prognostic model for hospital mortality at ICU admission. Intensive Care Med. 231:1345–1355. doi: 10.1007/s00134-005-2763-5 16132892 PMC1315315

[18] Avesani R, Gambini MG, Albertini G (2006) The vegetative state: a report of two cases with a long-term follow-up. Brain Inj. 20:333–338. doi: 10.1080/02699050500487605 16537275

[19] Beis JM, Seyer JL, Brugerolle B, Le Chapelain L, Thisse MO, Mainard D, et al (2009) Care protocol for persistent vegetative states (PVS) and minimally conscious state (MSC) in Lorraine: retrospective study over an 18-year period. Ann Phys Rehabil Med. 52:374–381. doi: 10.1016/j.rehab.2009.05.003 19541559

[20] Lin LC, Hsieh PC, Wu SC (2008) Prevalence and associated factors of pneumonia in patients with vegetative state in Taiwan. J Clin Nurs. 17(7):861–868. doi: 10.1111/j.1365-2702.2006.01883.x 17419784

[21] Ng YS, Chua KS (2005) States of severely altered consciousness: clinical characteristics, medical complications and functional outcome after rehabilitation. NeuroRehabilitation. 20:97–105. 15920302

[22] Seel RT, Douglas J, Dennison AC, Heaner S, Farris K, Rogers C (2013) Specialized early treatment for persons with disorders of consciousness: program components and outcomes. Arch Phys Med Rehabil. 94:1908–1923. doi: 10.1016/j.apmr.2012.11.052 23732166

[23] Katz DI, Polyak M, Coughlan D, Nichols M, Roche A (2009) Natural history of recovery from brain injury after prolonged disorders of consciousness: outcome of patients admitted to inpatient rehabilitation with 1–4 year follow-up. Prog Brain Res. 177:73–88. doi: 10.1016/S0079-6123(09)17707-5 19818896

[24] Lucca LF, Lofaro D, Pignolo L, Leto E, Ursino M, Cortese MD, et al (2019) Outcome prediction in disorders of consciousness: the role of coma recovery scale revised. BMC Neurol. 19:68. doi: 10.1186/s12883-019-1293-7 30999877 PMC6472098

[25] Chelly H, Bahloul M, Ammar R, Dhouib A, Mahfoudh KB, Boudawara MZ, et al (2019) Clinical characteristics and prognosis of traumatic head injury following road traffic accidents admitted in ICU “analysis of 694 cases” Eur J Trauma Emerg Surg. 45:245–253. doi: 10.1007/s00068-017-0885-4 29234838

[26] Jaul E, Calderon-Margalit R (2007) Persistent vegetative state and dementia in the elderly. Int Psychogeriatr. 19:1064–1071. doi: 10.1017/S104161020600473X 17234039

[27] Laureys S, Boly M (2007) What is it like to be vegetative or minimally conscious? Curr Opin Neurol. 20:609–613. doi: 10.1097/WCO.0b013e3282f1d6dd 17992077

[28] Geocadin RG, Koenig MA, Jia X, Stevens RD, Peberdy MA (2008) Management of Brain Injury After Resuscitation From Cardiac Arrest. Neurol Clin. 26(2): 487506. doi: 10.1016/j.ncl.2008.03.015 18514823 PMC3074242

[29] Oujamaa L, Francony G, Boucheix P, Schilte C, Bouzat P, Perennou D, et al (2017) Dynamics of clinical recovery during the early phase of rehabilitation in patients with severe traumatic and non-traumatic brain injury. Brain Inj. 31:1463–1468. doi: 10.1080/02699052.2017.1376759 28956630

[30] Adigüzel E, Yaşar E, Kesikburun S, Demir Y, Aras B, Safaz I, et al (2018) Are rehabilitation outcomes after severe anoxic brain injury different from severe traumatic brain injury? A matched case-control study. Int J Rehabil Res. 41:47–51. doi: 10.1097/MRR.0000000000000261 29200410

[31] Harbinson M, Zarshenas S, Cullen NK (2017) Long-Term Functional and Psychosocial Outcomes After Hypoxic-Ischemic Brain Injury: A Case-Controlled Comparison to Traumatic Brain Injury. PM R. 9(12):1200–1207. doi: 10.1016/j.pmrj.2017.04.015 28512065

[32] Sasannejad C, Wesley-Ely E, Lahiri S (2019) Long-term cognitive impairment after acute respiratory distress syndrome: a review of clinical impact and pathophysiological mechanisms. Crit Care. 23:352. doi: 10.1186/s13054-019-2626-z 31718695 PMC6852966

[33] Wannez S, Heine L, Thonnard M, Gosseries O, Laureys S, Coma Science Group collaborators (2017) The repetition of behavioral assessments in diagnosis of disorders of consciousness. Ann Neurol. 81:883–889. doi: 10.1002/ana.24962 28543735

[34] Matthews EA, Magid-Bernstein J, Sobczak E, Velazquez A, Falo CM, Park S, et al (2018) Prognostic Value of the Neurological Examination in Cardiac Arrest Patients After Therapeutic Hypothermia. Neurohospitalist. 8:66–73. doi: 10.1177/1941874417733217 29623156 PMC5882011

[35] Knapik P, Trejnowska E, Knapik M, Kręt M, Cieśla D, Krzych ŁJ, et al (2019) Young Adults Among Patients Admitted to Polish Intensive Care Units in the Silesian ICU Registry. Med Sci Monit. 25: 5727–5737. doi: 10.12659/MSM.913852 31371694 PMC6689200

[36] Kübler A, Siewiera J, Durek G, Kusza K, Piechota M, Szkulmowski Z (2014) Guidelines regarding the ineffective maintenance of organ functions (futile therapy) in ICU patients incapable of giving informed statements of will. Anaesthesiol Intensive Ther. 46:215–220. doi: 10.5603/AIT.a2014.0038 25293473

[37] Patel K, Sinvani L, Patel V, Kozikowski A, Smilios C, Akerman M, et al (2018) Do-Not-Resuscitate Orders in Older Adults During Hospitalization: A Propensity Score-Matched Analysis. J Am Geriatr Soc. 66:924–929. doi: 10.1111/jgs.15347 29676777

[38] Knapik P, Krzych ŁJ, Weigl W, Adamski J, Hultstöm M (2017) Mortality Rate in Polish Intensive Care Units Is Lower Than Predicted According to the APACHE II Scoring System. Intensive Care Med. 43:1745–1746. doi: 10.1007/s00134-017-4883-0 28733717 PMC5633622

[39] Alliprandini M, Ferrandin A, Fernandes A, Belim M, Jorge M, Colombo B, et al (2019) End-of-life management in intensive care units: a multicentre observational prospective cohort study. Anaesthesiol Intensive Ther. 51:348–356. doi: 10.5114/ait.2019.91189 31893601

[40] World Health Organization (2020) Covid-19 https://covid19.who.int/region/euro/country/pl Accessed 9 November 2020.

