# Supplementary material for: Epidemiology and clinical characteristics of patients discharged from the ICU in a vegetative or minimally conscious state
Source: PLoS One. 2021 Jun 25;16(6):e0253225. doi: 10.1371/journal.pone.0253225 (PMC8232456; doi:10.1371/journal.pone.0253225)
Supplement: S1 File — (DOC) [file pone.0253225.s001.doc]

## Supplement File 1. Details of the multivariate logistic regression model.

## Full-model

|  | OR | -95% CI | +95% CI | p |
| --- | --- | --- | --- | --- |
| Unconcious at admission (admission status) | 5,67 | 4,39 | 7,33 | **<0,001** |
| Cardiac arrest (primary cause of admission) | 4,19 | 3,58 | 4,89 | **<0,001** |
| Craniocerebral trauma (primary cause of admission) | 3,32 | 2,64 | 4,18 | **<0,001** |
| Chronic neurological disorders (comorbidities) | 1,64 | 1,33 | 2,03 | **<0,001** |
| Previous cerebral stroke (comorbidities) | 1,58 | 1,24 | 2,01 | **<0,001** |
| Disorders of consciousness (primary cause of admission) | 1,15 | 0,99 | 1,32 | **0,062** |
| Alcoholism (comorbidities) | 1,07 | 0,87 | 1,31 | 0,540 |
| Arterial hypertension (comorbidities) | 0,96 | 0,84 | 1,11 | 0,624 |
| Bacterial infection (primary cause of admission) | 0,93 | 0,75 | 1,15 | 0,499 |
| Heart failure (primary cause of admission) | 0,81 | 0,69 | 0,96 | **0,015** |
| Chronic renal failure (primary cause of admission) | 0,74 | 0,58 | 0,95 | **0,018** |
| Sepsis (primary cause of admission) | 0,65 | 0,41 | 1,04 | **0,072** |
| Exacerbation of respiratory failure (primary cause of admission) | 0,57 | 0,42 | 0,78 | **<0,001** |
| Acute respiratory failure (primary cause of admission) | 0,57 | 0,48 | 0,66 | **<0,001** |
| Postoperative (primary cause of admission) | 0,49 | 0,40 | 0,60 | **<0,001** |
| Acute pancreatitis (primary cause of admission) | 0,22 | 0,05 | 0,90 | **0,035** |
| Cancer (primary cause of admission) | 0,16 | 0,08 | 0,30 | **<0,001** |

Full-model statistics

|  | Stat. | Stat/Df |
| --- | --- | --- |
| Deviation | 6067,8 | 0,42 |
| Pearson Chi2 | 14797,3 | 1,03 |
| AIC | 6109,8 |  |
| AICC | 6109,9 |  |
| BIC | 6268,9 |  |
| R2 Coxa-Snella | 0,100 |  |
| R2 Nagelkerka | 0,244 |  |

Hosmer Lemeshow = 22,19, poziom p= 0,0046

# Reduced model

|  | OR | -95% CI | +95% CI | p |
| --- | --- | --- | --- | --- |
| Unconcious at admission (admission status) | 5,95 | 4,73 | 7,49 | **p<0.001** |
| Cardiac arrest (primary cause of admission) | 4,33 | 3,72 | 5,03 | **p<0.001** |
| Craniocerebral trauma (primary cause of admission) | 3,52 | 2,81 | 4,41 | **p<0.001** |
| Chronic neurological disorders (comorbidities) | 1,66 | 1,34 | 2,05 | **p<0.001** |
| Previous cerebral stroke (comorbidities) | 1,57 | 1,24 | 2,00 | **p<0.001** |
| Heart failure (primary cause of admission) | 0,80 | 0,68 | 0,94 | **0.007** |
| Chronic renal failure (primary cause of admission) | 0,73 | 0,57 | 0,93 | **0.012** |
| Exacerbation of respiratory failure (primary cause of admission) | 0,60 | 0,44 | 0,82 | **0.001** |
| Acute respiratory failure (primary cause of admission) | 0,59 | 0,51 | 0,69 | **p<0.001** |
| Postoperative (primary cause of admission) | 0,48 | 0,39 | 0,59 | **p<0.001** |
| Acute pancreatitis (primary cause of admission) | 0,21 | 0,05 | 0,88 | **0.033** |
| Cancer (primary cause of admission) | 0,16 | 0,08 | 0,30 | **p<0.001** |

Reduced model statistics

|  | Stat. | Stat/Df |
| --- | --- | --- |
| Deviation | 6086,2 | 0,42 |
| Pearson Chi2 | 14860,3 | 1,04 |
| AIC | 6112,2 |  |
| AICC | 6112,2 |  |
| BIC | 6210,6 |  |
| R2 Coxa-Snell | 0,099 |  |
| R2 Nagelkerke | 0,242 |  |

Hosmer Lemeshow = 23,45, p= 0,0028
